# A New Genotype of Feline Morbillivirus Infects Primary Cells of the Lung, Kidney, Brain and Peripheral Blood

**DOI:** 10.3390/v11020146

**Published:** 2019-02-09

**Authors:** Michael Sieg, Johannes Busch, Maria Eschke, Denny Böttcher, Kristin Heenemann, Annett Vahlenkamp, Anja Reinert, Johannes Seeger, Romy Heilmann, Kira Scheffler, Thomas W. Vahlenkamp

**Affiliations:** 1 Institute of Virology, Faculty of Veterinary Medicine, Leipzig University, An den Tierkliniken 29, 04103 Leipzig, Germany; Michael.Sieg@vetmed.uni-leipzig.de (M.S.); Johannes.Busch@vetmed.uni-leipzig.de (J.B.); Kristin.Heenemann@vetmed.uni-leipzig.de (K.H.); 2 Institute of Immunology, Faculty of Veterinary Medicine, Leipzig University, An den Tierkliniken 11, 04103 Leipzig, Germany; maria.eschke@uni-leipzig.de; 3 Institute of Pathology, Faculty of Veterinary Medicine, Leipzig University, An den Tierkliniken 33, 04103 Leipzig, Germany; denny.boettcher@vetmed.uni-leipzig.de; 4 Tierarztpraxis Böhlen Dr. A. Vahlenkamp, Leipziger Straße 73, 04564 Böhlen, Germany; info@tierarztpraxis-boehlen.de; 5 Institute of Veterinary Anatomy, Histology and Embryology, Faculty of Veterinary Medicine, Leipzig University, An den Tierkliniken 43, 04103 Leipzig, Germany; anja.reinert@vetmed.uni-leipzig.de (A.R.); seeger@vetmed.uni-leipzig.de (J.S.); 6 Department for Small Animals, Veterinary Teaching Hospital, Faculty of Veterinary Medicine, Leipzig University, An den Tierkliniken 23, 04103 Leipzig, Germany; romy.heilmann@kleintierklinik.uni-leipzig.de; 7 Institute for Medical Physics and Biophysics, Medical Department, Leipzig University, Härtelstraße 16-18, 04107 Leipzig, Germany; kira.scheffler@medizin.uni-leipzig.de

**Keywords:** cat, paramyxovirus, kidney disease, cell tropism, phylogeny

## Abstract

Paramyxoviruses comprise a large number of diverse viruses which in part give rise to severe diseases in affected hosts. A new genotype of feline morbillivirus, tentatively named feline morbillivirus genotype 2 (FeMV-GT2), was isolated from urine of cats with urinary tract diseases. Whole genome sequencing showed about 78% nucleotide homology to known feline morbilliviruses. The virus was isolated in permanent cell lines of feline and simian origin. To investigate the cell tropism of FeMV-GT2 feline primary epithelial cells from the kidney, the urinary bladder and the lung, peripheral blood mononuclear cells (PBMC), as well as organotypic brain slice cultures were used for infection experiments. We demonstrate that FeMV-GT2 is able to infect renal and pulmonary epithelial cells, primary cells from the cerebrum and cerebellum, as well as immune cells in the blood, especially CD4^+^ T cells, CD20^+^ B cells and monocytes. The cats used for virus isolation shed FeMV-GT2 continuously for several months despite the presence of neutralizing antibodies in the blood. Our results point towards the necessity of increased awareness for this virus when clinical signs of the aforementioned organs are encountered in cats which cannot be explained by other etiologies.

## 1. Introduction

Paramyxoviruses constitute a large family of single stranded, negative-sense, enveloped RNA viruses that can lead to important diseases in humans and animals [1]. Historically, *Paramyxoviridae* have been subdivided into seven genera based on biochemical properties and SDS-PAGE patterns of viral structural proteins: Rubulavirus, Avulavirus, Respirovirus, Henipavirus, Morbillivirus, Ferlavirus and Aquaparamyxovirus. Taking genome sequences and protein data into account many currently described paramyxoviruses are assigned as unclassified, e.g. rodent-borne Tailam Virus [2], Nariva Virus [3] and Bank Vole Virus [4], as well as paramyxoviruses detected in bats [5]. 

In recent years, the genus morbillivirus has received growing attention, due to the discovery of a new feline morbillivirus (FeMV, formerly abbreviated as FmoPV) associated with tubulo-interstitial nephritis in stray cats from Hong Kong [6]. Subsequently, the prevalence was reported from other countries including Japan, USA, Turkey, Brazil, Thailand, Italy and Germany [7,8,9,10,11,12,13]. Percentage of FeMV-positive urines ranged from 3% to 23% in the US [8] and Japan [14], respectively. Seroprevalence data of FeMV available from Hong Kong and Japan showed 27.8% [6], 23.1% [14], 21.0% [15] and 22% [16] of investigated cats to be FeMV-positive using nucleo- or phosphoproteins as antigens.

While some of these studies established a link between an infection with FeMV and the presence of kidney diseases in affected cats [6,7,12,13,15], others could not confirm such an association [8,9,10,14]. These discrepancies may be due to the complexity of chronic kidney disease (CKD) pathogenesis in general, making it difficult to link cases of feline CKD to only one specific trigger [17]. In some cats, feline morbilliviruses may induce a persistent infection of the urinary tract [8]. So far it is not clear whether an acute or chronic infection can cause or support the development of CKD. During our current studies an unknown feline paramyxovirus was detected in urine samples from domestic cats [13]. Although this virus was initially linked to FeMV strains from Japan, whole genome sequencing revealed a different genotype of FeMV, tentatively named feline morbillivirus genotype 2 (FeMV-GT2). Here we show that the FeMV-GT2-Gordon strain replicates in primary feline epithelial cells from different organs and is able to infect primary feline T and B cells, as well as monocytes in vitro. We demonstrate that FeMV-GT2 readily infects feline organotypic brain slice cultures with cells of the cerebrum and cerebellum being comparably susceptible. 

The molecular and biological characterization of FeMV-GT2 shows that the diversity of feline paramyxoviruses extends beyond the formerly known FeMV isolates, which must be further studied in detail.

## 2. Materials and Methods 

### 2.1. Cell Culture

All cell lines and primary cells used were maintained at 37 °C, 90% humidity and 5% CO_2_. LLC-MK2 and Vero CCL81 cell lines were purchased from the Instituto Zooprofilattico Sperimentale della Lombardia e dell’Emilia Romagna «Bruno Ubertini» (IZSLER), Italy, whereas CrFK, MDBK, MDCK-II, HEK 293, BHK-21, MA-104, MARC-145, A9, FMN-R, MGN-R, RAN-2-R, FLN-R and KE-R were kindly provided by the Friedrich-Loeffler-Institute (FLI), Germany. All cell lines were grown in Dulbecco’s Modified Eagle Medium (DMEM) containing 4.5 g/L glucose, 5% FBS, GlutaMAX™ supplement, 1 × MEM non-essential amino acids solution and 1 mM sodium pyruvate. Fcwf­4 cells (ATCC^®^ CRL2787™) were purchased from the American Type Culture Collection (ATCC), USA and cultivated in RPMI 1640 medium containing 2 mM L-glutamine, 1.5 g/L sodium bicarbonate, 4.5 g/L glucose, 10 mM HEPES, 1.0 mM sodium pyruvate, 0.05 mM 2-mercaptoethanol, 10% FBS or in DMEM with 10% FBS, respectively.

### 2.2. Isolation of Primary Feline Cells 

The organ material used in this work was provided by the Institute of Pathology, Faculty of Veterinary Medicine, Leipzig University and derived from dead animals euthanized for medical reasons unrelated to this study. 

Primary feline kidney cells were isolated by adapting a previously described protocol [18]. Briefly, kidneys from dead animals were removed aseptically and stored in ice cold Hank’s buffered salt solution (HBSS) without CaCl_2_ and MgCl_2_ until further processing. Kidneys were de-capsulated, bisected, and the renal cortex was removed and cut into small pieces. Tissue was dissociated by collagenase II (1 mg/mL) treatment at 37 °C for 30 min. This step was repeated three times and the collected cell suspensions were passed through a 100 µm cell strainer to remove cell aggregates. Cells were pelleted by centrifugation at 400× *g* for 10 min and re-suspended in complete culture medium (DMEM:F12 [50%:50%] medium containing 10 ng/mL human Epidermal Growth Factor, 50 nM hydrocortisone, 5 µg/mL insulin, 5 µg/mL transferrin, 50 nM sodium selenite and 100 IU/mL penicillin-streptomycin) and seeded into 75 cm^2^ plastic flasks. The cells were cultured at 37 °C under 5% CO_2_ in a humidified atmosphere until the monolayer became nearly confluent. Similarly, primary feline urinary bladder cells were prepared by collagenase treatment of the urinary bladder mucosa. Dissociated cells were than cultured in keratinocyte serum free medium (KSFM) including 5 ng/mL EGF, 0.2% (*v*/*v*) of bovine pituitary extract (BPE, Thermo Fisher) and 30 ng/mL Choleratoxin (Sigma-Aldrich, St. Louis, MO, USA) and 100 IU/mL penicillin-streptomycin.

To isolate primary feline pulmonary epithelial cells fresh lungs were rinsed three times with DMEM through the primary bronchus to remove alveolar macrophages. Pulmonary epithelial cells were dissociated from the surrounding tissue with 0.05% trypsin and 0.02% EDTA followed by an incubation for 30 min at 37 °C. Finally, cells were collected by rinsing the lungs with DMEM and a centrifugation step for 10 min at 500× *g*. Cells were seeded into a 75 cm^2^ plastic flask and cultured in DMEM containing 4.5 g/L glucose, 5% FBS, GlutaMAX™ supplement, 1 × MEM non-essential amino acids and 1 mM sodium pyruvate, 100 IU/mL penicillin-streptomycin until the monolayer reached approximate confluence.

### 2.3. Collection of Samples, Virus Isolation and Virus Infection Assays

Urine samples from cats were collected as part of the routine physical examination at the Department of Small Animals, Leipzig University. The material was stored at −20 °C (for not more than 4 weeks) before RNA isolation was conducted. 

For virus isolation Vero, CrFK and LLC-MK2 cells were infected with 1 ml urine mixed with 5 mL pure DMEM (containing 100 IU/mL penicillin-streptomycin) in 75 cm^2^ cell culture flasks. After 24 h incubation at 37 °C, 5% CO_2_ and 90% humidity the inoculum was replaced by 8 mL cultivation medium (DMEM, sodium pyruvate, non-essential amino acids, 2% FBS, 100 IU/mL penicillin-streptomycin) and were cultured for 6 days at the indicated conditions. The cell culture supernatant from this first passage was used to infect fresh cells. This procedure was repeated several times and the resulting cell culture supernatants were continuously tested for the presence of paramyxovirus-RNA by RT-PCR. For virus infection assays different cell lines (see Section 2.1) were infected with the LLC-MK2-adapted (passage no. 3) ‘Gordon’ strain of FeMV-GT2 in a 24-well format at an MOI ~ 0.1 for two hours at 37 °C. Infection medium was replaced by cultivation medium (see above) and cells were further incubated at 37 °C, 5% CO_2_ and 90% humidity for five days. Percentage of virus-positive cells were semi-quantified by immunofluorescence staining targeting the FeMV-GT2-nucleoprotein followed by microscopic analysis.

### 2.4. Electron Microscopy

Viral particles were concentrated from cell culture supernatants of FeMV-GT2-infected LLC-MK2 via ultracentrifugation. In brief, 10 ml virus-containing supernatant was sublayered with 2 mL of a 20% sucrose cushion and centrifuged using an SW32Ti rotor (Beckman Coulter) at 30,000 rpm for 1.5 h at 4 °C. The semi-purified virions were re-suspended in PBS prior to loading onto Formvar-coated copper grids (Plano GmbH, Wetzlar, Germany) and were negatively stained with 2% uranyl acetate. Grids were viewed using an TEM Libra 120 (Carl Zeiss, Jena, Germany) transmission electron microscope.

### 2.5. Detection, Genome Sequencing and Phylogenetic Analysis

Detection of FeMV-GT2 in feline urine samples or in cell culture supernatants was performed as previously described [13,19] using the primer pair HEN-RES-MOR-R and HEN-RES-MOR-F1/F2. For whole genome sequencing, degenerated primers were designed based on the available FeMV-sequences (primer sequences are available in supplementary files, Table S1). Amplification was performed with the “SuperScript™ III One-Step RT-PCR System containing Platinum™ Taq High Fidelity DNA Polymerase” (Thermo Fisher Scientific, Waltham, MA, USA). PCR products were visualized by agarose gel electrophoresis with 1× Tris-acetate-EDTA buffer (40 mM tris-acetate, 1 mM EDTA, pH 8.3) containing 0.2 µg/mL of ethidium bromide. For DNA-sequencing, specific PCR fragments were cut out of the gel and purified using a Gel/PCR DNA Fragments Extraction Kit (Geneaid, New Taipei City, Taiwan). Sequencing was performed by Sanger’s dideoxy termination method (Microsynth Seqlab GmbH, Göttingen, Germany) and each PCR product was sequenced twice. Nucleotide sequences were analyzed using the Basic Local Alignment Search Tool (BLAST, www.ncbi.nlm.nih.gov/blast/), and genetic distances of whole genome sequences were calculated applying the general time reversible model with gamma distributed invariant sites (GTR + I) at the nucleotide level using the MEGA 6 software. A phylogenetic tree was built by the maximum likelihood method with 1000 bootstrap replicates [20]. Sequences determined in the course of this work have been deposited in GenBank, with the accession numbers MK182089 and MK182090.

### 2.6. Cell Infection Assays and Immunofluorescence Staining

Cells were fixed with 2% (*w*/*v*) paraformaldehyde for 10 min at 4 °C, permeabilized with 0.1% Triton X-100 in PBS for 5 min, blocked with 3% BSA-PBS for 30 min at room temperature and probed with a cytokeratin pan type I/II antibody cocktail (clone AE1/AE3, Thermo Fisher Scientific, Waltham, MA, USA) at a dilution of 1:1000. FeMV-GT2 was probed using a polyclonal rabbit antibody raised against the nucleoprotein of FeMV (generated in house) at a dilution of 1:100. After overnight incubation at 4 °C cells were washed three times with PBS and incubated with an Alexa Fluor 488-conjugated goat-anti-mouse IgG (H+L) or with an Alexa Fluor 546-conjugated goat-anti-rabbit IgG (H+L) secondary antibody (Thermo Fisher Scientific, Waltham, MA, USA) in 3% BSA-PBS at a dilution of 1:1000 containing 4′,6-diamidino-2-phenylindole (DAPI, 300 nM) and incubated for one hour at 37 °C in a humidified chamber. After three PBS-washing steps images were acquired using an AX70 microscope (Olympus, Tokio, Japan). Virus titration was performed by the endpoint dilution assay in LLC-MK2 cells followed by visualization of virus positive dilutions using immunofluorescence staining. Viral titers were expressed as 50% tissue culture infective dose (TCID_50_) calculated after the Reed–Muench method [21].

### 2.7. Neutralization Assay

To test for FeMV-GT2-neutralizing antibodies a serum neutralization tests (SNT) was established. Briefly, cell monolayers of LLC-MK2-cells were used at approximately 90% of confluence in a 96-well microtiter format. Feline serum samples were incubated at 56 °C for 30 min to inactivate complement components followed by the preparation of two-fold serial dilutions in pure DMEM starting with a four-fold dilution. Each sample was then was mixed with ~200 infectious viral particles (TCID_50_) of FeMV-GT2-Gordon strain in an equal volume of DMEM followed by an incubation period of one hour at 4 °C. This suspension was used for infection of LLC-MK2-cells for two hours at 37 °C. Samples were then replaced by DMEM including 2% FBS, sodium pyruvate, non-essential amino acids and 100 IU/mL penicillin-streptomycin. Four days later, viral plaques were visualized applying the described immunofluorescence protocol (see Section 2.6). Each experiment was performed in duplicates. Neutralizing titers were defined as the reciprocal of the serum dilution at which the number of plaques was reduced to 50 % relative to virus-only controls.

### 2.8. Infection of Feline PBMC

Feline blood samples were obtained from healthy cats by venipuncture into vacutainer tubes containing EDTA as an anticoagulant (BD Vacutainer^®^, 10 mL, Beckton Dickinson, Heidelberg, Germany). Peripheral blood mononuclear cells (PBMC) were isolated as described previously by using Histopaque-1077 (Sigma-Aldrich, St. Louis, MO, USA) [22]. Freshly isolated cells were resuspended in RPMI 1640 medium (without supplements) and infected with FeMV-GT-Gordon strain, MOI = 0.1 for one hour at 37 °C. The infection medium was removed by centrifugation at 500 × *g* for 5 min, cells were re-suspended and further cultured for 48 h at 37 °C in a 24-well cell culture plate (Greiner bio-one, Kremsmünster, Austria) at a density of 1 million cells per ml. RPMI 1640 medium supplemented with 10% FBS, 1 mM sodium pyruvate, 1% (*v*/*v*) non-essential amino acids and 10 IU/mL recombinant human interleukin-2 (Thermo Fisher Scientific, Waltham, MA, USA) was used for cultivation. Mock-infected PBMC from each animal served as negative controls and were treated similarly. After the indicated incubation period cells were centrifuged at 500 × *g* for 5 min and the cell pellet washed twice with PBS. For discrimination of dead cells in the consecutive flow cytometric analysis, FeMV-GT2- and mock-infected PBMC were stained with fixable viability dye eFluor780 (Thermo Fisher Scientific, Waltham, MA, USA) according to the manufacturer’s protocol. Subsequently, cells were incubated with 10% (*v*/*v*) goat normal serum (Thermo Fisher Scientific, Waltham, MA, USA) for 15 min on ice to avoid non-specific Fc binding. Staining of surface markers was performed using FITC-anti-cat CD4 (clone vpg34, BIO-RAD, Herkules, CA, USA), Alexa Fluor^®^ 647-anti-human CD20 (clone 2H7, BIO-RAD, Herkules, CA, USA) and PE-anti-human CD8 (clone 3B5, Thermo Fisher Scientific, Waltham, MA, USA). The corresponding isotype controls were all purchased from eBioscience. Surface staining was performed by incubation of cells with fluorochrome-conjugated antibodies in FACS-buffer (PBS with 3% FBS) for 15 min on ice. Stained cells were fixed for 20 min at 4 °C with 2% (*w*/*v*) paraformaldehyde. For intracellular detection of FeMV-GT2, cells were permeabilized using 0.5% saponin in FACS-buffer for 20 min at room temperature (RT). The virus was labeled by incubation with a rabbit polyclonal anti-FeMV-nucleoprotein antibody (generated in-house) at 1 µg/mL (in FACS-Saponin-buffer for 20 min at room temperature followed by two washing steps in FACS-saponin buffer. Finally, cells were incubated with a 1:100 dilution of Pacific Blue™-anti-rabbit antibody (Thermo Fisher Scientific, Waltham, MA, USA) in FACS-saponin buffer for 20 min at RT followed by two consecutive washing steps with FACS buffer. The cells were acquired with a BD LSR Fortessa^TM^ flow cytometer (Becton Dickinson, Heidelberg, Germany) and viable cells were analyzed using the FlowJo 7.6.5 software (Treestar Inc., Ashland, OR, USA). The gating strategy is shown in supplementary files, Figure S1. 

### 2.9. Infection of Feline Organotypic Brain Slice Cultures with FeMV-GT2 

To investigate neuronal tropism of FeMV-GT2 we established an in vitro model of the feline brain by generating organotypic slice cultures from cerebrum and cerebellum. Brains from animals euthanized for medical reasons unrelated to this study, were dissected and the cerebrum was separated from the cerebellum. Following preparation the tissue was immediately submerged in ice-cold HBSS and slices were generated using a VT1000s (Leica, Wetzlar, Germany) microtome. Due to the difference in tissue rigidity, slices of the cerebrum were cut to 200 µm and of the cerebellum were cut to 300 µm thickness. Freshly prepared brain slices were transferred to cell culture inserts (0.4 µm, Merck Millipore) pre-equilibrated with HBSS and placed into 6-well cell culture plates (Greiner bio-one, Austria). For cultivation, minimum essential medium (MEM) containing 25% (*v*/*v*) heat-inactivated horse serum, 10 mM L-glutamine, 25 mM HEPES, 25 mM HBSS and 10 IU/mL penicillin-streptomycin was used and changed daily. Cultures were incubated at 37 °C, 90% humidity and 5% CO_2_. After 17 days of cultivation each slice was infected by direct application of 1800 FFU of FeMV-GT2-Gordon strain to the tissue. Negative controls (mock infections) were generated by applying an equivalent volume of DMEM with 2% FBS to the brain tissue. 10 days after infection slices were washed with PBS and fixed with 4% (*w*/*v*) paraformaldehyde for 60 min. Slices were permeabilized with 0.2% Triton X-100 in PBS (TPBS) for 30 min and blocked in TPBS with 20% horse serum and 2% BSA for 60 min. The primary antibody raised against the FeMV-nucleoprotein was diluted (1:100) in blocking buffer and incubated with the samples for 72 h at 4 °C with constant agitation. After washing with PBS a goat anti-rabbit IgG (H+L) cross-adsorbed, Alexa Fluor^®^ 488 labeled secondary antibody (Thermo Fisher Scientific, Waltham, MA, USA) was diluted (1:1000) in blocking buffer, applied to the slices and incubated for two hours. Nuclei were counter-stained using DAPI (300 nM). After two subsequent washing steps with PBS the slices were visualized using a Leica SP 8 confocal microscope.

## 3. Results

### 3.1. Detection, Isolation and Culture of FeMV-GT2

As part of the German surveillance program for paramyxoviruses in domestic cats, 723 urine samples from animals were analyzed using the described paramyxovirus consensus RT-PCR [19]. Six samples (0.83%) were positive for a previously unknown paramyxovirus, tentatively named feline morbillivirus genotype 2 (FeMV-GT2). Based on physical examination and urinalysis findings all six animals were diagnosed with diseases of the urinary tract (i.e., acute or chronic kidney disease; Table 1). No viral RNA was detectable within the available serum and EDTA-blood samples from these urine-positive animals. Two cats (‘Gordon’ and ‘TV25’) were sampled for several months and were found to be positive for FeMV-GT2-RNA by PCR within the urine at all time points tested (Table 1). Electron microscopy analysis and successful virus isolation from fresh urine samples of these two animals revealed that intact viral particles were released. Interestingly, corresponding serum samples from ’Gordon’ and ‘TV25’ showed high titers of FeMV-GT2-specific neutralizing antibodies (titer > 256). 

**Figure 1:**
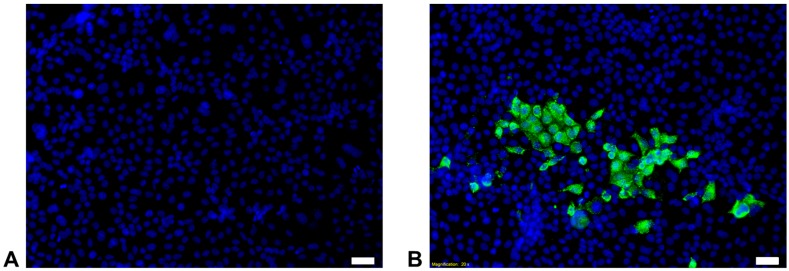
Isolation of FeMV-GT2-Gordon strain on LLC-MK2 cells. Five days *post infection* passage no. 3 was stained with a FeMV-nucleoprotein-specific antibody (shown in green). Images were taken at 200-fold magnification. Cell nuclei are stained in blue. (**A**) Mock infection; (**B**) Infection with FeMV-GT2. Scale bars represent 20 µm.

To characterize this virus further, isolation attempts from fresh urine samples of affected animals (appropriate samples were only available from the animals ‘Gordon’ and ‘TV25’) were conducted using Vero, CrFK and LLC-MK2 cells. After the third passage cell culture supernatants were tested for the presence of paramyxoviruses using RT-PCR. We successfully isolated FeMV-GT2 strains from both animals. The results revealed that all three cell lines were susceptible to FeMV-GT2 with LLC-MK2 cells being more permissive in comparison to Vero and CrFK cells. These findings were also confirmed by immunofluorescence staining targeting the FeMV-GT2-nucleoprotein (Figure 1). No visible CPE was induced on any tested cell line. Initial virus titer on LLC-MK2 cells was low at passage no. 3 (3 × 10^3^ TCID_50_/mL), but could be enhanced to 1 × 10^5^ TCID_50_/mL after passage no. 9. Virus titer remained low (~1 × 10^3^ TCID_50_/mL) on CrFK and Vero cells independent from the passage number (tested until passage no. 8). Both virus strains, FeMV-GT2-Gordon and FeMV-GT2-TV25, exhibit similar growth kinetics. As a consequence, virus stock was produced on LLC-MK2 cells and was used for further infection experiments. 

To exclude contamination with other feline viruses, viral stock was concentrated via ultracentrifugation and tested for feline Herpesvirus (FeHV), feline Coronavirus (FCoV), feline Calicivirus (FCV) and feline Parvovirus (FPV) using specific PCR systems [23,24,25,26]. None of the aforementioned viruses were found to be present in the FeMV-GT2 preparations.

### 3.2. FeMV-GT2 Virions Display a Morphology Typical for Paramyxoviruses

**Figure 2:**
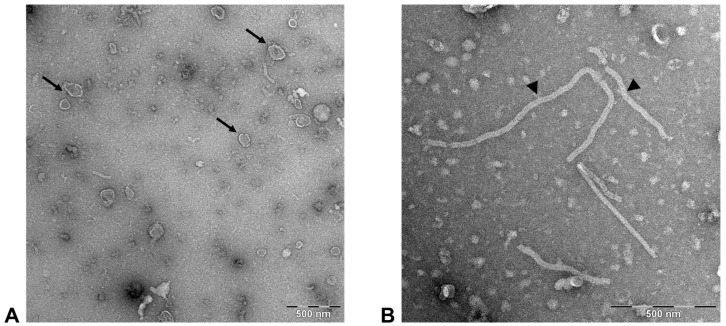
Electron microscopy of FeMV-GT2. Cell culture supernatant of FeMV-GT2-infected LLC-MK2 cells was concentrated via ultracentrifugation and virions were negatively stained using uranyl acetate. (**A**) typical pleomorphic paramyxovirus particles (arrows); (**B**) free nucleocapsids (arrowheads). Scale bar represents 500 nm.

To visualize FeMV-GT2 virions, cell culture supernatants were semi-purified and examined by transmission electron microscopy. The observed viral particles showed typical paramyxoviral morphology represented by pleomorphic, enveloped virions sized between 100–200 nm (Figure 2A). 

In addition, characteristic free nucleocapsids were found in all virus preparations varying between 25–35 nm in diameter (Figure 2B). No differences in viral morphology were detected between samples originating from urine or from cell culture supernatants.

### 3.3. FeMV-GT2 Replicates in Kidney Cell Lines from Simian, Feline and Rodent Origin

To investigate the in vitro growth spectrum of FeMV-GT2, several cell lines of different animal origin were tested for their susceptibility and efficiency for virus production. Experiments were done with the LLC-MK2-adapted FeMV-GT2-Gordon strain at passage no. 3. As summarized in Table 2, Fcwf-4 and LLC-MK2 cells were most susceptible to FeMV-GT2, with ~ 75% of the cells being FeMV-GT2 positive by IFA five days after infection. CrFK, Vero, MARC-145 and BHK-21 were also susceptible, but to a lesser extent (Table 2). To exclude the possibility that these observations were the result of an adaption phenomenon from the three passages on LLC-MK2 cells, the original urine sample from ‘Gordon’ was applied to the selected cell lines and analyzed for the permissiveness regarding FeMV-GT2. No differences between the urine sample and the LLC-MK2-adapted FeMV-GT2-Gordon strain were observed. Due to the better growth and the easier maintenance of LLC-MK2 cells in comparison to the Fcwf-4 cell line, all further FeMV-GT2 strains were isolated and propagated on the former cell line. In contrast to other known morbilliviruses, no cytopathic effects became apparent on any of the FeMV-GT2-susceptible cell lines (tested until passage number five).

### 3.4. FeMV-GT2 Differs from Classical Feline Morbillivirus Strains Genetically 

In order to determine the phylogenetic relationship between already known feline morbilliviruses and the isolated strains from this study, whole genome amplification was performed. Primers were designed from conserved regions of available FeMV sequences. FeMV-GT2 fragments were amplified and gaps were filled by applying the primer walking strategy. Near-complete genome sequences of two FeMV-GT2 strains (‘Gordon’ and ‘TV25’, accession numbers MK182089 and MK182090) from domestic cats were revealed. Nucleotide sequences from strains were 99.2% identical, differing in only 130 nucleotides mainly located in the non-coding regions of the genome. The genome organization of FeMV-GT2 resembled that of classical FeMV strains, encoding six (3′-N-P/V/C-M-F-H-L-5′) open reading frames (ORFs), typical for other morbilliviruses. Pairwise alignments of the ORFs between FeMV and FeMV-GT2 demonstrated nucleotide identities ranging between 80.5% (phosphoprotein) and 83.3% (matrix protein) and an overall whole genome nucleotide identity of about 78.2% (Table 3). Amino acid conservation between FeMV and FeMV-GT2 was found to be higher than 85% for all structural proteins, except for the phosphoprotein where the amino acid identity of the FeMV-GT-Gordon strain to FeMV was only 75% (Table 3). 

**Figure 3:**
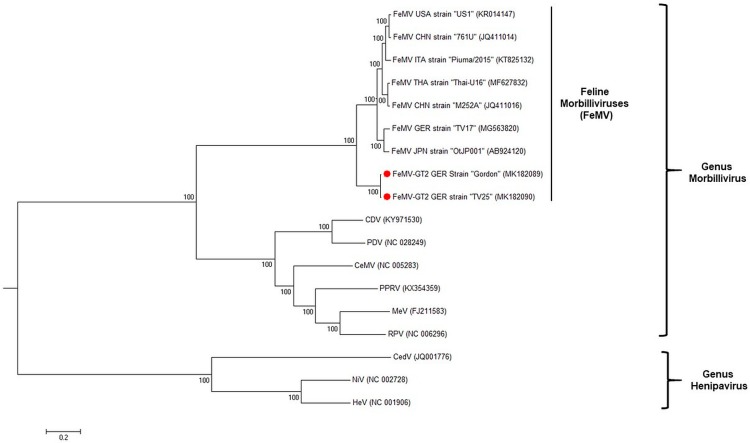
Phylogenetic tree of paramyxoviruses constructed from whole genome sequences. Strains identified in this study were highlighted in red. Scale bar indicates 0.2 nucleotide substitutions per site. Accession numbers of paramyxovirus species included in the phylogenetic analysis are shown in brackets. Abbreviations: CDV = canine distemper virus, PPRV = peste-des-petits-ruminants virus, MeV = measles virus, RPV = rinderpest virus, CeMV = cetacean morbillivirus, PDV = phocine distemper virus, CedV = Cedar Virus, NiV = Nipah Virus, HeV = Hendra Virus.

Phylogenetic analysis based on whole genome sequences demonstrated that FeMV-GT2 is genetically closely related to (Figure 3), but yet distinct from, previously described feline morbilliviruses [6]. Therefore, we suggest that FeMV-GT2 should be considered as a different genotype of feline morbilliviruses. 

### 3.5. Primary Epithelial Cells from the Feline Kidney and Lung as Wells as Immune Cells are Susceptible to FeMV-GT2 Infection 

To elucidate the target tissues of FeMV-GT2 we established protocols for the isolation of primary feline cells from various organs of cats (see Section 2.2). Experimental in vitro infection was performed using the LLC-MK2-adapted FeMV-GT2-Gordon strain. We initially investigated primary cells from the urinary tract and successfully established a method for the preparation and cultivation of feline primary kidney cells as described in materials and methods. Isolated cells represented a mixture (approximately 50%:50%) of cytokeratin-positive epithelial cells and vimentin-positive non-epithelial cells. An in-depth analysis further showed that cells also expressed the renal collecting duct cell marker aquaporin-2 [27], the distal tubulus cell marker calbindin D28K [28] and the proximal tubulus cell marker alkaline phosphatase [29]. Hence, it was concluded that the isolated primary feline kidney cells express the terminal differentiation markers also present in naïve tissues and are therefore suitable for in vitro infection experiments. The cells were infected at an MOI of 0.5 for two hours and thereafter cultured for five days prior to FeMV-GT2 nucleoprotein staining. Mock-infected cells served as negative controls. 

**Figure 4:**
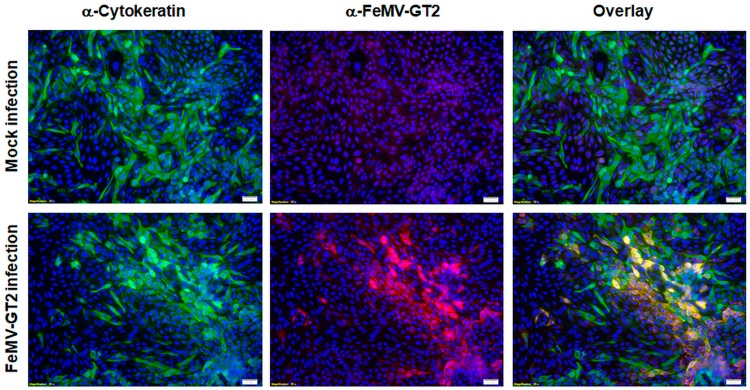
Susceptibility of primary feline kidney cells to FeMV-GT2. Cells were inoculated with FeMV-GT2-Gordon strain (MOI = 0.5) or mock-infected, fixed five days p.i. and stained for cytokeratin (shown in green) or FeMV-GT2 (shown in red). Double positive cells are orange colored. Cell nuclei were visualized with DAPI (shown in blue). Images were taken at 200-fold magnification. Scale bars represent 20 µm.

We observed approx. 25% of primary feline kidney cells to be FeMV-GT2 positive, whereas no signal was detected in the negative control (Figure 4), showing that primary feline kidney cells were susceptible to FeMV-GT2 infection in vitro. No cytopathic effects (e.g. formation of syncytia) or significant cell death in comparison to the mock-infection was detected. These observations were reproduced from cell preparations of three individual animals. The nature of the FeMV-GT2-susceptible cell types was elucidated by cytokeratin staining (marker for cells of epithelial origin). Renal epithelial cells were shown to be the main target of FeMV-GT2 (Figure 4), although a minority (~ 10%) of non-epithelial cells were also susceptible to this virus. 

**Figure 5:**
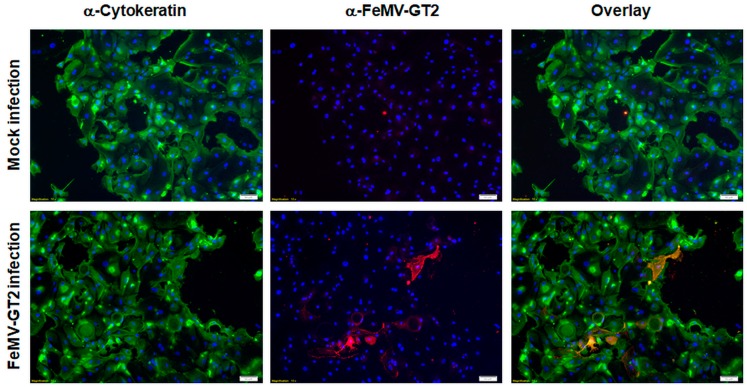
Susceptibility of feline bladder epithelial cells to FeMV-GT2. Primary feline urinary bladder cells were inoculated with FeMV-GT2-Gordon strain (MOI = 0.5) or mock-infected, fixed five days p.i. and stained for cytokeratin (shown in green) or FeMV-GT2 (shown in red). Double positive cells are depicted in orange. Cell nuclei were visualized with DAPI (shown in blue). Images were taken at 100-fold magnification. Scale bars represent 50 µm.

To elucidate the involvement of adjacent organs in virus shedding, primary feline bladder epithelial cells were isolated and infected with FeMV-GT2 as described above. As shown in Figure 5, primary feline bladder cells were successfully isolated from clinical material and stained positive for cytokeratins as expected for cell types of epithelial origin. Urinary bladder cells were shown to be viable, due to trypan blue exclusion, and due to the possible cultivation for at least three passages. In contrast to kidney cells, only a few urinary bladder cells were susceptible to FeMV-GT2. This finding was reproduced from isolated urinary bladder epithelial cells of different animals (*n* = 3). 

**Figure 6:**
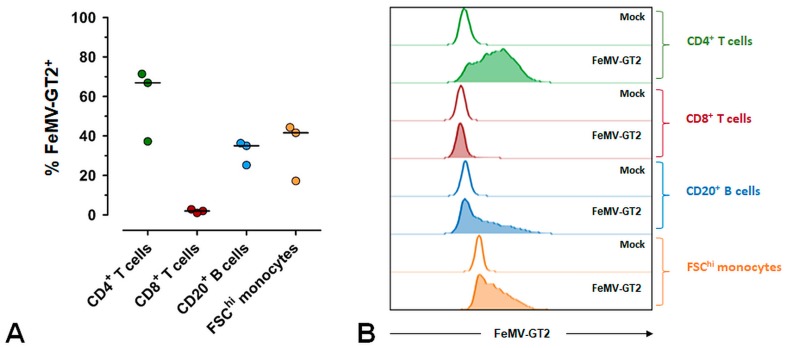
Susceptibility of different blood cell populations to FeMV-GT2. PBMC from healthy cats were infected with FeMV-GT2-Gordon strain (MOI = 0.1) or mock-infected. After 48 hours FeMV-GT2-infected cells among CD4^+^ T cells, CD8^+^ T cells, CD20^+^ B cells and FSC^hi^ monocytes were analyzed by flow cytometry. Only viable cells following doublet exclusion were included in the analysis. Fluorescence-minus-one (FMO) controls were used for appropriate gating of CD4^+^ T cells, CD8^+^ T cells and CD20^+^ B cells. See supplemental Figure S1 for gating strategy. (**A**) Frequencies of FeMV-GT2-positive cells among CD4^+^ T cells, CD8^+^ T cells, CD20^+^ B cells and FSC^hi^ monocytes of feline peripheral blood are depicted. Gates were set according to the respective mock control. Each dot represents one individual cat, the horizontal bars indicate median values. Data from one representative experiment (three independent experiments were performed) are shown. (**B**) Histograms show representative results of FeMV-GT2-infected PBMC populations in comparison to mock controls.

To determine the capability of FeMV-GT2 to infect feline immune cells we isolated PBMC from healthy cats and performed in vitro infection experiments. Cats were proven to be FeMV- and FeMV-GT2-negative by immunofluorescence analysis of serum samples and by RT-PCR analysis of corresponding urine samples. Intracellular virus in different immune cell populations was quantified 48 h after infection by flow cytometry (Figure 6). The results of these investigations demonstrated that CD4^+^ T cells, CD20^+^ B cells and FSC^hi^ monocytes are susceptible to FeMV-GT2 infections in vitro. The percentage of virus-positive cells varied from 40–70% for CD4^+^ T cells and from 20–40% for both CD20^+^ B cells and FSC^hi^ monocytes (Figure 6A). In contrast, CD8^+^ T-cells were less susceptible to FeMV-GT2 (Figure 6B) with percentages of virus positive cells varying between 2–5%. In addition, flow-cytometric analysis of FeMV-GT2 infected PBMC revealed that about 15% of non-T and non-B lymphocytes were also positive for FeMV-GT2. These cell types likely represent natural killer (NK) cells, but due to a lack of specific antibodies to phenotype feline NK-cells this could not be confirmed.

**Figure 7:**
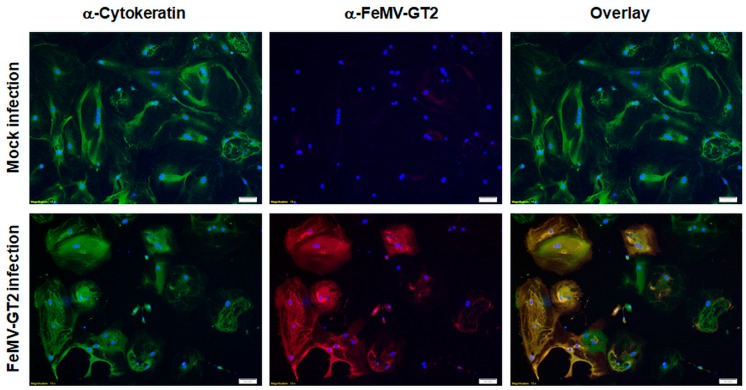
Susceptibility of feline pulmonary epithelial cells to FeMV-GT2. Primary feline pulmonary epithelial cells were inoculated with FeMV-GT2-Gordon strain at an MOI of 0.5 or mock-infected. Cells were fixed five days p.i. and stained for cytokeratin (shown in green) or FeMV-GT2 (shown in red). Double positive cells are shown in orange. Cell nuclei were visualized with DAPI (shown in blue). Images were taken at 100-fold magnification. Scale bar represents 50 µm.

Due to the fact that other morbilliviruses infect epithelial cells of the lung, feline primary pulmonary epithelial cells were also used for FeMV-GT2 infection experiments. Cells were probed with a pan-cytokeratin antibody to discriminate cells of epithelial origin, alveolar macrophages and fibrocytes. Representative results of distinct experiments are shown in Figure 7. 

No contaminating alveolar macrophages or fibrocytes were detected in these cell preparations via IFA analysis. It can be stated that the vast majority of the cultured cells represented pulmonary epithelial cells. These primary epithelial cells were infected with FeMV-GT2-Gordon strain at an MOI of 0.5 for two hours and further cultured for five days before analysis. The experiments revealed that about 70–85% of the cultured pulmonary epithelial cells were susceptible to FeMV-GT2. All virus-positive cells were also cytokeratin-positive. No cytopathic effect (e.g. formation of syncytia) or significant cell death was observed.

### 3.6. Organotypic Brain Slice Cultures from the Feline Cerebrum and Cerebellum are Susceptible to FeMV-GT2 Infection In Vitro

**Figure 8:**
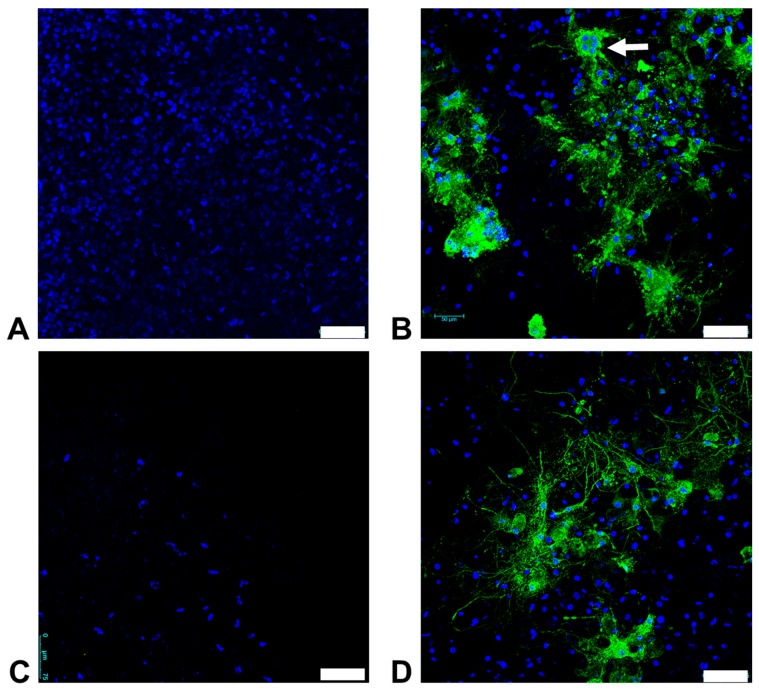
Susceptibility of feline organotypic brain slice cultures to FeMV-GT2. A representative staining result from the cerebellum (A and B) and from the Cerebrum (C and D) is shown. After 17 days in culture the slice was infected with 1800 FFU of FeMV-GT2-Gordon strain or mock-infected. At day 10 p.i. slices were fixed and stained with an α-FeMV-nucleoprotein antibody (shown in green). Cell nuclei were stained with DAPI (shown in blue). Note the formation of small syncytia in the cerebellum (white arrow), (**A**,**C**) Mock infection; (**B**,**D**) FeMV-GT2-infection. Scale bars indicate 75 µm.

We were able to cultivate brain slice cultures from adult cats for at least four weeks. When infecting these cultures with FeMV-GT2-Gordon strain, it became evident that both the cerebrum and cerebellum were highly susceptible (Figure 8). 

Interestingly, infected slice cultures of the cerebellum displayed small syncytia (5–10 nuclei per cell) (Figure 8B). This finding was the first evidence that FeMV-GT2 can induce cytopathic effects to particular cell types as it is known for other paramyxoviruses. In contrast, CPE was not visible in FeMV-GT2-infected slice cultures of the cerebrum (Figure 8D). FeMV-GT2-infected cells from the cerebrum and the cerebellum showed large amounts of nucleoprotein in the entire cell body and cell protrusions. All infected cells retained a typical morphology of brain cells. Virus-positive cells grouped together in distinctive patterns so that virus transmission via cell-to-cell contact might have occurred. All described observations were verified on at least three slice cultures from the cerebrum and the cerebellum. The specific nature of the susceptible cell types (e.g. neurons, astroglia and microglia) could not be determined definitely, but staining of the brain slice cultures with a GFAP-specific antibody (glial fibrillary acidic protein, marker for glia cells) showed no co-localization of FeMV-GT2-positive cells with GFAP-positive structures. Neurons could not be stained, due to the lack of specific feline antibodies.

## 4. Discussion

The family of *Paramyxoviridae* is currently divided into seven distinct genera. The number of unassigned paramyxoviruses is still increasing because well-established classification schemes, like sequence comparisons based on the viral RNA-dependent RNA polymerase (vRdRp) or pairwise analysis of sequence complementarity (PASC) of all ORFs is often connected to several difficulties among new paramyxoviruses [30]. Feline morbilliviruses (FeMV) were found to be clearly distinguishable from the classical morbilliviruses, such as CDV or MeV based on phylogenetic analyses [14,15] despite the fact that the size and organization of their genome reflects properties as defined for the genus morbilliviruses. Here, we describe a second morbillivirus isolated from domestic cats, tentatively named feline morbillivirus genotype 2 (FeMV-GT2). This virus grouped together with previously described feline morbilliviruses (FeMV), but could be separated from these isolates based on whole genome sequences (Figure 3). Amino acid identities between the FeMV-GT2 and the FeMV structural proteins ranged between 75–92%. These differences are comparable to the species cetacean morbillivirus (CeMV) where currently five strains from different animal species exhibit a similar genetic diversity as identified for FeMV and FeMV-GT2 in our study [31]. In addition, electron microscopy revealed a similar morphology of the FeMV-GT2 particle as described for FeMV [14] and other morbilliviruses. We observed a wider ribonucleocapsid protein (RNP) in FeMV-GT2 preparations, but this could be due to a preparation or staining artefact, but may also be an intrinsic feature of feline morbilliviruses. Therefore, we provisional propose that FeMV-GT2 should be considered as a different genotype of FeMV. To clarify the taxonomic status of FeMV-GT2 more information about the genetic diversity of circulating strains is needed and should be addressed to future studies.

The prevalence of FeMV-GT2 was low in urine (0.83%), but this may be influenced by the storage conditions. Urine samples were collected as part of the routine clinical work and for practical reasons they were stored for some weeks at -20 °C before RNA extraction could be performed. Thus, RNA degradation may have occurred influencing the sensitivity of the PCR analysis. Two cats were FeMV-GT2-positive for several months till years, with virus shedding in their urine emphasizing that the virus is able to establish persistent infections. This is in accordance with published data about prolonged FeMV-infections in domestic cats [8,13,32]. Interestingly, virus secretion within the urine in two persistently infected cats (‘Gordon’ and ‘TV25’, Table 1) continued despite high titers of FeMV-GT2-specific neutralizing antibodies in the blood. This is in contrast to other morbillivirus infections were neutralizing antibody titers higher than 32 or 120 prevent virus secretion in CDV [33] and MeV [34], respectively. The reason for this phenomenon remains speculative at the moment, but can be the result of FeMV-GT2 replication in epithelial cells of the kidney, FeMV-GT2 escape mutants or impaired T-cell response in infected animals. 

All FeMV-GT2-positive cats were affected with diseases of the urinary tract, but the number of positive cases was too small to suggest the virus as the cause of the observed illnesses. An alternative explanation could be that FeMV-GT2 can only replicate on already damaged tissues of the urotract. In addition, it is known that CKD is common in geriatric cats with prevalence ranging between 28–50% [35,36] leading to the possibility of a statistical connection between FeMV-GT2 and CKD. To further elucidate whether FeMV-GT2 infection is a cause or effect of acute and/or chronic urotract diseases, controlled animal infection experiments are required. 

FeMV-GT2 was propagated in various kidney cell lines from primate, rodent and feline origin although no CPE was observed in any of the tested cell lines. This is in contrast to published results from FeMV as this virus is able to induce syncytia formation in CrFK cells [14,32]. These discrepancies could be due to lower viral titers (~10-fold) of FeMV-GT2 in comparison to FeMV [14,32] or may be a result of the viral adaption process to cell line not capable of expressing the wild type entry receptor which might be necessary for inducing cytopathic effects as described for MeV [37]. 

We showed that feline primary kidney cells are susceptible to FeMV-GT2 infection in vitro and that epithelial cells are the primary target of FeMV-GT2 in our in vitro model. Whether this virus location is also reflected in natural infections needs to be clarified by (immune)-histopathology in diseased or experimentally infected cats. Feline urinary bladder epithelial cells were significantly less susceptible to FeMV-GT2. The reproducibility of the results excludes the possibility that this observation was caused by the cat’s individual medical histories. Data generated from in vitro infection experiments might, however, differ from the in vivo situation. The cultivation medium used for primary kidney cells, for instance, might influence the expression of terminal differentiation markers and serial passaging of these cells can induce dedifferentiation of primary distal renal tubular cells [38]. Equally, primary bladder epithelial cells might not display their physiological surface markers, so-called uroplakins, when cultured in vitro [39]. Therefore, it is possible that the FeMV-GT2 tissue tropism observed in our in vitro experiments differs from the in vivo conditions. On the other hand, FeMV was shown to replicate in renal tubular cells in naturally infected cats [6,15,40], supporting our results for a kidney-associated FeMV-GT2 replication. 

Although persistent infections with FeMV-GT2 are evident, none of the analyzed blood samples harbored detectable amounts of viral RNA. This implicates that viremia is present—if at all—only for a short period of time. This observation is supported by previous investigations in FeMV-infected cats where viremia was not detectable in the vast majority of blood samples [6,7,9]. There is only one report from a shelter in Thailand that found FeMV to be present in the blood of animals [11]. Currently, the mode of transmission of feline morbilliviruses is unknown. Related viruses (e.g. canine distemper and measles virus) establish infections in their susceptible hosts by transmission via the oro-nasal route. Following an initial replication in resident immune cells (tissue macrophages or dendritic cells) these viruses infect different lymphocyte subtypes resulting in a short-term viremic state [37]. Here we show that FeMV-GT2 can infect several cells of the immune system in the peripheral blood including CD4^+^ T cells, B cells, monocytes and to a significantly lesser extent CD8^+^ T cells. Feline PBMC were shown to be susceptible to FeMV-GT2 infection without prior stimulation supporting the assumption that the in vitro results reflect the in vivo situation. Percentage and identity of FeMV-GT2-positive immune cells resemble findings from in vivo infection experiments with CDV in a ferret model [41]. Similar results were also observed in a macaque model of MeV-infections [42]. Recently it was also shown that FeMV can be detected in the spleen and lymph nodes in naturally infected cats [40]. 

The in vitro infection experiments using primary feline pulmonary epithelial cells, as well as cells from the cerebrum and the cerebellum revealed that they are susceptible to FeMV-GT2. Whether alveolar macrophages may also contribute to virus replication in the lung could not be investigated in this study, due to the lack of specific antibodies for phenotyping feline alveolar macrophages. Alongside infections of the lung, CDV and MeV are able to infect and induce severe damage to the brain under certain circumstances [37]. Although the mechanism of how CDV and MeV enter the brain is not completely understood, a leukocyte-associated virus transport as a source of hematogenous brain infections is hypothesized [37]. Our in vitro findings show that such a mechanism might also be responsible for the spread of FeMV-GT2 in the body of affected animals as brain slice cultures were susceptible to the FeMV-GT2-Gordon strain. Although the specific nature of the affected cell types could not be determined definitely, it is likely that neurons are a target as GFAP-positive cells (representing glia cells) were found to be FeMV-GT2-negative in our in vitro experiments. These data revealed that FeMV-GT2 is able to infect feline brain cells integrated into a physiological tissue environment. However, this needs further investigation in controlled animal infection experiments. 

Although the observed in vitro tropism of FeMV-GT2 is in line with naturally FeMV-infections [6,15,40] and resembles the pathology of other morbilliviruses as CDV or MeV [37] it cannot be completely excluded that these are in part the result of viral mutations emerged through the cell culture adaption process. It is well known that Vero-adapted MeV strains acquired mutations leading to the usage of CD46 as a viral entry receptor which is not a characteristic of MeV wild type strains [43]. The possibility that FeMV-GT2 have gained mutations might explain our observation that viral titer increased about 100-fold on LLC-MK2 cells between consecutive passages. On the other hand, titers remained low in CrFK, Vero and BHK-21 cells independent from the number of blind passages. Therefore, raising titers in the first passages of LLC-MK2 cells can also be addressed to a low concentration of augmentable virus in urine samples. In depth sequencing approaches of FeMV-GT2-positive urine samples and the respective cell culture adapted viral isolates will help to interpret our in vitro findings in the context of viral pathogenesis and should be conducted in future studies. 

Nevertheless, clinicians should be aware of FeMV-GT2 when respiratory or neurological signs are encountered. Although the aforementioned organs are well known as infections sites for CDV and MeV [37], they have not been characterized as target tissues for FeMV in the past. Until recently, the feline kidney was focused on being the target of feline morbilliviruses [6,7,8,9,10,11,12,13,14,15,16]. Our data clearly demonstrate that FeMV-GT2 can infect primary cells obtained from other organs and that the pathologic potential of FeMV and FeMV-GT2 might currently be underestimated. Hence, further research is needed to elucidate the impact of feline morbilliviruses on animal health, especially for domestic cats. 

## 5. Conclusions

In summary, we isolated and characterized a new genotype of feline morbillivirus (FeMV-GT2) and showed the in vitro tropism to primary feline cells from the kidney, lung and the immune system, as well as to organotypic slice cultures from the feline cerebrum and cerebellum. Our results indicate that veterinarians should be aware of this virus when symptoms of the aforementioned organs are encountered which cannot be explained by other reasons. 

## 6. Patents

Part of the work reported in this manuscript resulted in the patent entitled “New Paramyxovirus and uses thereof” with the international application no.: PCT/EP2017/071392.

## Figures and Tables

**Table 1:** Characteristics of FeMV-GT2-positive cats.

Animal ID	Sex	Age (in Years)	Clinical Diagnosis	Duration of FeMV-GT2 Shedding in Urine	nAb-titer Against FeMV-GT2
90604	male	18	CKD (IRIS stage 2, normotensive), pancreatitis	n.a.^1^	n.a.^2^
93436	female	15	Hypertension, renal cyst, lipiduria	n.a.^1^	n.a.^2^
98450 (‘Gordon’)	male	13	CKD (IRIS stage 2, normotensive, non-proteinuric)	>6 months	>256
TV25	male	6	non-obstructive FLUTD, proteinuria, lipiduria	>2 years	>256
118649	female	6	diabetic ketoacidosis, cholangitis, pancreatitis, azotemia	n.a.	n.a.^2^
118650	female	13	CKD, feline triaditis, sepsis, consumption coagulopathy	n.a.^1^	n.a.^2^

Abbreviations: CKD = chronic kidney disease; FLUTD = feline lower urinary tract disease; IRIS = International Renal Interest Society; n.a. = not available; ^1^ = no follow-up urine samples were available due to euthanasia, ^2^ = no serum sample available, nAb = neutralizing antibodies.

**Table 2:** Growth spectrum of FeMV-GT2-Gordon strain in different cell lines.

Cell line	Tissue	Species	Growth of FeMV-GT2 *
Fcwf-4	Whole fetus, macrophage	*Felis catus*	+ + +
CrFK	Kidney, epithelial	*Felis catus*	+ +
KE-R	Embryo, fibroblastic	*Felis catus*	-
LLC-MK2	Kidney, epithelial	*Macaca mulatta*	+ + +
Vero (CCL81)	Kidney, epithelial	*Chlorocebus pygerythrus*	+
MA-104	Kidney, epithelial	*Macaca mulatta*	-
MARC-145	Kidney, epithelial	*Macaca mulatta*	+
BHK-21	Kidney, fibroblastic	*Mesocricetus auratus*	+
A9	Adipose tissue, fibroblastic	*Mus musculus*	-
HEK-293	Kidney, epithelial	*Homo sapiens*	-
MDBK	Kidney, epithelial	*Bos taurus*	-
MDCK-II	Kidney, epithelial	*Canis lupus familiaris*	-
FMN-R	Kidney, epithelial	*Microtus arvalis*	-
MGN-R	Kidney, fibroblastic	*Myodes glareolus*	-
RAN-2-R	Kidney, epithelial	*Rousettus aegyptiacus*	-
FLN-R	Kidney, epithelial	*Eptesicus serotinus*	-

* Susceptibility of represented cell lines to FeMV-GT2-Gordon strain infection was semi-quantified after five days p.i. using immunofluorescence staining against FeMV-GT2-nucleoprotein: + + + = ~ 75% FeMV-GT2-positive cells, + + = ~ 50% FeMV-GT2-positive cells, + = ~ 25% FeMV-GT2-positive cells, - = 0% FeMV-GT2-positive cells.

**Table 3:** Whole genome comparison between FeMV and FeMV-GT2-Gordon strain *.

Genom Region	Nucleotide Position	Nucleotide Homology to FeMV ‘M252A’	Amino Acid Identity to FeMV ‘M252A’
3‘-untranslated region	1–107	84.1%	not applicable
Nucleocapsid-protein	108–1667	81.1%	89.8%
Intergenic region	1668–1780	39.8%	not applicable
Phospho-protein	1781–3256	80.5%	75.1%
Intergenic region	3257–3388	44.7%	not applicable
Matrix-protein	3389–4402	83.3%	91.7%
Intergenic region	4403–4949	48.6%	not applicable
Fusion-protein	4950–6581	80.9%	88.7%
Intergenic region	6582–6958	56.0%	not applicable
Hemagglutinin-protein	6959–8746	80.5%	85.9%
Intergenic region	8747–8887	54.6%	not applicable
Polymerase-protein	8888–15496	82.5%	90.9%
5‘-untranslated region	15497–16047	52.4%	not applicable
Whole genome	1–16047	78.2%	not applicable

* Whole genome of the FeMV-GT2-Gordon strain is compared to the prototype FeMV strain ‘M252A’ (Accession no.: JQ411016) from Hong Kong.

## Supplementary Materials

The following are available online at http://www.mdpi.com/1999-4915/11/2/146/s1, Table S1: Primer sequences used for the amplification of nearly whole genome sequences of FeMV-GT2 strains. Figure S1: Gating strategy used in flow cytometric analyses of PBMC.
