# Supplementary material for: A New Genotype of Feline Morbillivirus Infects Primary Cells of the Lung, Kidney, Brain and Peripheral Blood
Source: Viruses. 2019 Feb 9;11(2):146. doi: 10.3390/v11020146 (PMC6410220; doi:10.3390/v11020146)
Supplement: Supplementary file 1 [file viruses-11-00146-s001.pdf]

Supplementary Figure S1. Gating strategy used in flow cytometric analyses of PBMC.

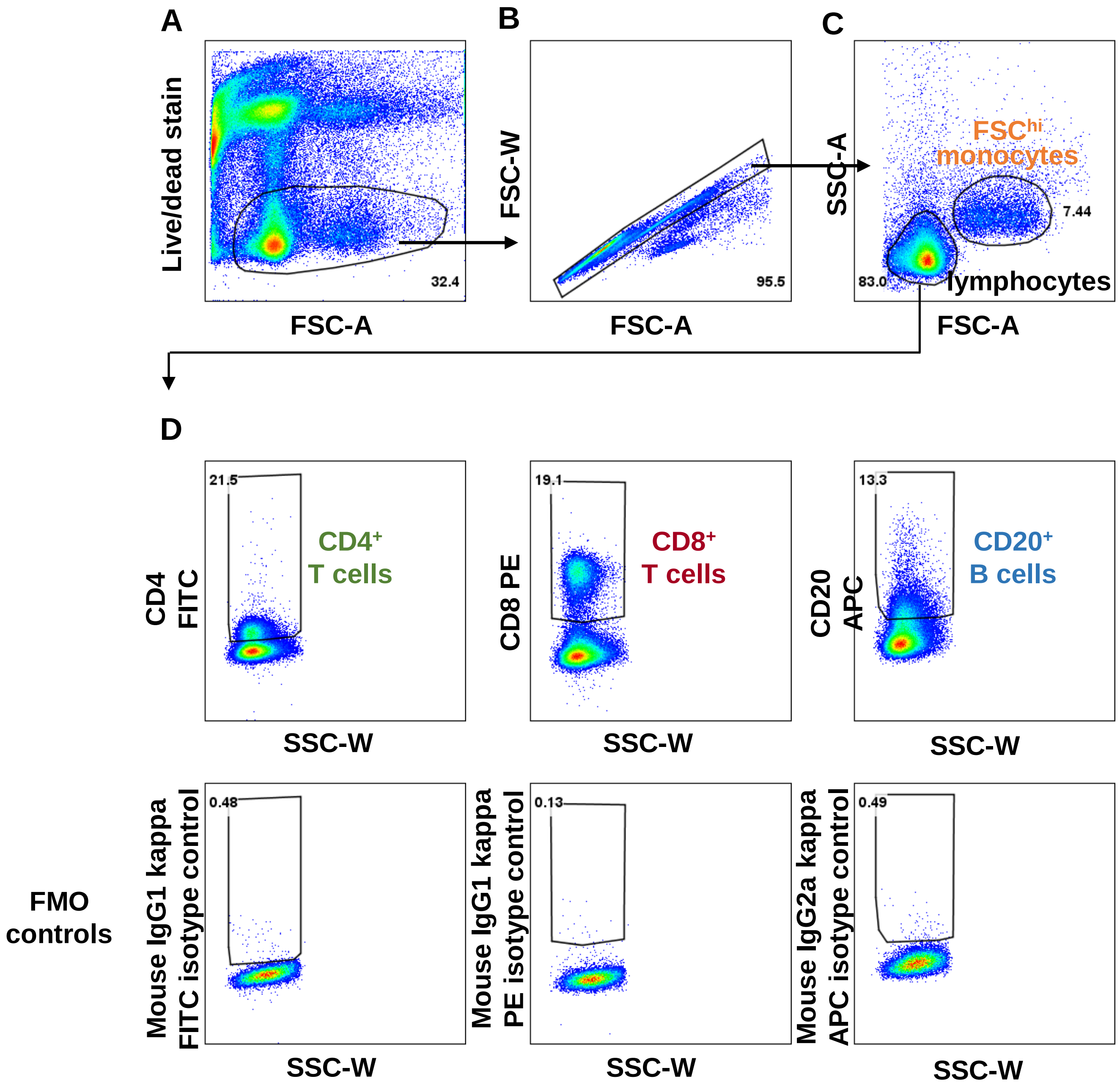

Legend to Figure S1: Gating strategy used in flow cytometric analyses of PBMC.

Representative pseudocolor plots are shown. After exclusion of dead cells (**A**) and doublets (**B**), gating on lymphocytes and monocytes was performed according to their forward and side scattering (FSC/SSC) properties (**C**). (**D**) Among lymphocytes $CD4^+$ T cells, $CD8^+$ T cells and $CD20^+$ B cells (upper panel) were gated according to the appropriate Fluorescence-minus-one (FMO) controls (lower panel).

Table S1. Primer sequences used for the amplification of nearly whole genome sequences of FeMV-GT2 strains.

Primer name	Nucleotide sequence (5' → 3')	Size of PCR fragment	Position (GenBank Acc.-No. MK182089)
Gordon3'Ende400bpFor<br>Gordon3'Ende400bpRev	ACCAACCTCAGGAACCACCT<br>ACCAGACAAAGATGTCTGTGACC	400 bp	1 – 434
NP-Schmusi-For<br>NP-TV2-Rev	ATGTCGAGTCTACTGAGGTCACTTGC<br>GATTTCAGAAGGTCAGTATCATTGTAATGG	1560 bp	108 – 1667
Phospho-Gordon-For<br>Phospho-Gordon-Rev	ATGTCAGCAGAACAAATCCAACAAG<br>GTTATTATTCTTGATCAATATCATAACTTTCTC	1500 bp	1781 – 3256
Gordon-InterReg3kbFor<br>GordonInterReg3kbRev	TTGAGCAGGTAATGGTTCATTG<br>CTTTCCCTTTAGGCGTAGGG	3000 bp	3612 – 6894
Gordon-InterReg1kbFor<br>GordonInterReg1kbRev	ATCGCACTGGAGTTTCTCTTG<br>TTGCCACTCCAGTCTTACAGG	1000 bp	4581 – 5490
Gordon2000bp-For<br>Gordon2000bp-Rev	GTCGGTAAGCCAGGGTTGTA<br>TGAAGTCATTGTCGGGGATA	2000 bp	8335 – 10346
Gordon6200bp-For<br>Gordon6200bp-Rev	CAAGACCTATCCCAGGCAAA<br>CCAATCCCAAAGTTGCTTGT	6200 bp	2384 – 8636
Gordon-InterReg5kbFor<br>GordonInterReg5kbRev	TCCAAGTACCATTCGCACAG<br>CCGACCAAACATGTTGCTAA	5000 bp	3002 – 7761
HA-Gordon-For<br>HA-Gordon-Rev	CAGCGGAAGGACTACTCTTAAT<br>ATTACAGTTTATTAAGTTGAAAATGGCAG	1800 bp	7138 – 8715
Gordon-PAR-For<br>Gordon-HEN-Rev	ATGCCTCCCAATGGTGC<br>TGCAAGGACATGCAATTTTT	1300 bp	10044 – 11738
Gordon-Poly628bp-For<br>Gordon-Poly628bp-Rev	TGCAACACTTCAGCCATCTG<br>GAGTGGCACGCTATACAACAA	630 bp	12852 – 13460
Gordon-Poly2100bp-For<br>Gordon-Poly2100bpRev	AGCCTCGGATCAACTAGCAA<br>AGCAATTGCAGTGACCAAAA	2100 bp	11260 – 13361
Gordon244bp-For<br>Gordon244bp-Rev	GTATCAATATTAAAGATCCAGGTAGTCTT<br>TACCCAGTCTTACATGAGAGTCAG	250 bp	15191 – 15433
Gordon5'Ende800bpFor<br>Gordon5'Ende800bpRev	ACCAGACAAAGAAAGCTATAGGTCC<br>CAGGTGCATTGCATTGAAGT	820 bp	15229 – 16047
